# Validation of the Integrated Care for Older People Screening Tool: Focus on the Chair Rise Test to Assess Locomotion

**DOI:** 10.1093/geroni/igab046.277

**Published:** 2021-12-17

**Authors:** Emmanuel Gonzalez-Bautista, Philipe de Souto Barreto, Aaron Salinas-Rodriguez, Sandrine Sourdet, Yves Rolland, Leocadio Rodriguez-Mañas, Sandrine Andrieu, Bruno Vellas

**Affiliations:** 1 Toulouse University Hospital (CHU Toulouse), Toulouse, Midi-Pyrenees, France; 2 CHU Toulouse: Centre Hospitalier Universitaire de Toulouse, Gérontopôle de Toulouse, Institut du Vieillissement, Midi-Pyrenees, France; 3 National Institute of Public Health of Mexico, Cuernavaca, Morelos, Mexico; 4 Getafe University Hospital, Getafe, Madrid, Spain; 5 University of Toulouse III, INSERM, UPS, Toulouse, Midi-Pyrenees, France

## Abstract

The Integrated Care for Older People (ICOPE) is a function- and person-centered healthcare pathway developed by the World Health Organization (WHO). ICOPE's first step (Step 1) consists of screening for impairments in the intrinsic capacity (IC) domains (namely sensorial, cognition, nutrition, psychological, and locomotion). For instance, the ICOPE Step1 tool suggests a cut-point of 14 seconds for five-repetition chair rise time as a marker of impaired locomotion. Given the lack of validation of this tool in the literature, we aimed to validate the ICOPE screening tool concerning incident health outcomes, focusing on the locomotion assessment. First, we analyzed the five-domain screening tool's ability to identify older adults (OA) at higher risk of incident outcomes (frailty, disability, dementia) using longitudinal data from the Multidomain Alzheimer Preventive Trial (MAPT). For the locomotion assessment (chair rise test), we derived and cross-validated age-specific cut points from two population-based cohorts using ROC (receiver operating characteristic) analysis. We further verified those cut points among OA real-life users of the health system and clinical trial participants. In conclusion, the ICOPE Step 1 screening tool was able to identify OA at higher risk of incident frailty, disability, and dementia. New chair-rise-time cut points for age groups 70-79 years old and 80 years and older were valid in populations from different settings. The ICOPE Step 1 tool provides a practical and integrative way of screening older adults for impairments in IC and detecting those at higher risk of functional decline.

